# Opn3 Drives Blue-Light-Induced Reduction in Lipid Droplets and Antiviral Defense

**DOI:** 10.3390/biom16010109

**Published:** 2026-01-08

**Authors:** Qifan Wu, Huiping Liu, Hongcui Liang, Xinyi Jiang, Yingqiao Qin, Shaomei Liang, Jingjing Wang, Kunpeng Liu

**Affiliations:** Guangxi Key Laboratory of Special Biomedicine, School of Medicine, Guangxi University, Nanning 530004, China; 2328391047@st.gxu.edu.cn (Q.W.); gxulhp123456@st.gxu.edu.cn (H.L.); 2337030222@st.gxu.edu.cn (H.L.); 2437030125@st.gxu.edu.cn (X.J.); 2237030141@st.gxu.edu.cn (Y.Q.); 2237030137@st.gxu.edu.cn (S.L.); 2437030117@st.gxu.edu.cn (J.W.)

**Keywords:** Opn3, lipid droplet degradation, Pparα, autophagy, antiviral therapy

## Abstract

Abnormal lipid metabolism is a key feature of many diseases. Therefore, investigating its underlying mechanisms is of great importance. Recently, blue light has shown promise as a drug-free way to influence energy metabolism, relying on the light-sensitive protein Opsin 3 (Opn3). This study aimed to investigate the effects of blue light irradiation on lipid droplet degradation in cells and its molecular mechanism, while also evaluating its potential antiviral effects. The results demonstrate that exposure to 470–480 nm blue light significantly reduced oleic-acid-induced intracellular lipid droplet accumulation and decreased triglyceride and total cholesterol levels, an effect dependent on the Opn3. It was found that blue light affects the Pparα signaling pathway through Opn3, and, at the same time, blue light and Opn3 promote autophagy mediated by p62 protein, thereby cooperatively regulating lipid droplet degradation. In Opn3 knockout cells, blue-light-induced lipid droplet degradation, nuclear accumulation of Pparα, and autophagic effects were all suppressed. Additionally, the study unexpectedly observed that blue light, via Opn3, significantly suppressed the replication of VSV, H1N1 and EMCV and alleviated virus-induced cell death and inflammatory responses. This study reveals the critical role of the blue light–Opn3-Pparα/p62 axis in regulating lipid droplet degradation in hepatocytes and identifies a novel antiviral function of Opn3-mediated blue light exposure. These findings provide a new theoretical basis and potential targets for innovative therapeutic strategies against metabolic diseases and viral infections.

## 1. Introduction

Lipid metabolism includes the synthesis, storage, transport, and breakdown of lipids within cells—processes that are vital for maintaining human health [1]. The degradation of lipids, a key part of this system, occurs mainly through lipolysis, lipophagy, and β-oxidation. Central to this process are lipid droplets (LDs), which serve as the main intracellular storage organelles for neutral lipids such as cholesteryl esters and triglycerides. They fulfill essential cellular metabolic requirements including lipid storage, energy metabolism, fatty acid transport, and signal transduction [2]. Dysregulated lipid metabolism contributes to various metabolic disorders including obesity, type 2 diabetes, fatty liver disease, and cardiovascular diseases [3,4,5], which pose serious threats to human health. Therefore, investigating the different pathways of intracellular lipid droplet degradation is of great importance.

Photo-biomodulation therapy (PBMT) is a technique that uses light radiation from visible to near-infrared wavelengths to influence biological tissues. Due to its non-invasive nature, safety, portability, ease of use, and low cost, PBMT is being increasingly adopted in clinical practice. Compared to traditional red light therapy, blue light is gaining attention for its potential in photo-biomodulation. Research showed that blue light in the 400–470 nm range has demonstrated intrinsic antibacterial properties [6], which can disrupt bacterial cell membranes and is utilized in treating acne. In human dermal fibroblasts, blue light exposure has been observed to reduce cell proliferation and metabolic activity [7], and high doses can also decrease fibroblast migration [8], suggesting potential benefits for managing psoriasis [9]. Additionally, Bauer J found that blue light serves as a primary treatment for neonatal jaundice, where it breaks down bilirubin through a photochemical reaction to alleviate symptoms [10]. Furthermore, low-dose 465 nm blue light has been shown to suppress the proliferation and migration of papillary thyroid carcinoma cells [11]. Importantly, the biological effects of blue light are not isolated events; rather, they are closely associated with cellular photoreceptors, such as opsin 3 (Opn3).

Opn3 is a light-sensitive protein located on the cell membrane. It acts as a non-visual photoreceptor by detecting light in tissues beyond the eyes [12]. This protein is highly expressed in the brain and testes [13] and is also found in other organs including the liver, heart, kidneys, and pancreas [14]. Meanwhile, Opn3 exhibits sensitivity to blue light around 480 nm [15]. Upon light stimulation, it activates intracellular signaling pathways, enabling it to mediate a range of non-visual, light-induced physiological processes. For instance, one study reported that accelerated wound healing following blue light exposure may be linked to a corresponding increase in Opn3 expression [16]. In another context, blue light activation of Opn3 triggers a calcium-dependent signaling cascade. This leads to MITF phosphorylation, which in turn increases the levels of tyrosinase and dopachrome tautomerase, thereby promoting melanin synthesis [17]. Interestingly, Opn3 also appears to play a regulatory role in adipose tissue. Research by Nayak et al. demonstrated that in mouse fat cells, Opn3 activation by blue light promotes lipolysis, a process that involves the accelerated phosphorylation of hormone-sensitive lipase. Furthermore, mice lacking the Opn3 gene showed a reduction in thermogenesis, indicating its importance in heat production [15].

Peroxisome proliferator-activated receptor alpha (Pparα) is a nuclear receptor that plays a central role in fatty acid metabolism. It is predominantly expressed in tissues like the liver and kidneys. Studies have shown that Pparα plays a significant role in numerous physiological processes, such as improving metabolic dysfunction-associated steatotic liver disease (MASLD) [18], enhancing immune responses in immune cells [19,20].

However, the mechanism by which blue light and Opn3 regulate lipolysis remains poorly understood. It is unclear whether they participate in lipid droplet degradation and how their action relates to classical lipid metabolic pathways. Using AML12 mouse hepatocytes, this study examined the impact of 470–480 nm blue light on the degradation of lipid droplets that had been accumulated through oleic acid (OA) treatment. Blue light exposure significantly reduced lipid droplet content and decreased triglyceride (TG) and total cholesterol (TC) levels in an Opn3-dependent manner. Opn3 KO cells confirmed that Opn3 is required for blue light-induced lipid droplet degradation. Furthermore, we identified critical roles for Pparα and p62 in this pathway: Pparα KO markedly inhibited blue light-mediated lipid droplet degradation, while p62 deletion blocked both Lc3b conversion and lipid droplet clearance. Given the significant impact of lipids on viral infection [21,22], we unexpectedly found that blue light exposure reduced replication of VSV, H1N1, and EMCV viruses, an effect abolished in Opn3 KO cells.

These results indicate that blue light promotes lipid droplet degradation through the Opn3- Pparα signaling axis, with the autophagy protein p62 playing an essential role. Additionally, Blue light combined with Opn3 reduces viral replication. This study advances our understanding of the relationship between light exposure and lipid droplet metabolism, offering new perspectives for treating related metabolic disorders and providing a foundation for antiviral research.

## 2. Methods

### 2.1. Antibodies and Reagents

Sodium oleate (S104196) was purchased from Sigma-Aldrich (St. Louis, MO, USA), and Oil Red O (O0625) was purchased from Sigma (Shanghai, China). BODIPY 493/503 (C2053S) and BSA (ST025) was purchased from Beyotime (Shanghai, China). Propidium Iodide (40710ES03) was purchased from Yeasen (Shanghai, China). Propidium iodide was purchased from Kunchuang Biotechnology (Xi’an, China).

The antibodies used in our experiments included: β-Actin (GB15001, Servicebio, WuHan, China), OPN3 (DF4877, Affinity, Cincinnati, OH, USA), PPARα (GB11163, Servicebio), LC3B (18725-1-AP, Proteintech, Chicago, IL, USA), P62 (18420-1-AP, Proteintech), H1N1 (11675-T62, Sino Biological, Beijing, China).

### 2.2. Cell Culture and Exposure Conditions

Mouse normal liver cells (AML12) and Human Non-Small Cell Lung Cancer Cells (A549) were cultured in high-glucose DMEM medium (G4515, Servicebio) supplemented with 10% fetal bovine serum (F101-01, Vazyme, Nanjing, China), 1% penicillin/streptomycin (PB180120, Pricella, Wuhan, China), and 10% insulin-transferrin-selenium medium supplement (ITSS-10201, OriCell, Guangzhou, China). The cells were cultured at 37 °C with 5% CO_2_ until 70–80% confluence was reached, then the experiment was initiated. Subsequently, the cells were exposed to 0.4 mM Oleic acid for 6 h before undergoing light exposure.

The blue LED light (470–480 nm) was purchased from Boxing Technology Co., Ltd. (Shenzhen, China). Cells were exposed to blue light with 0.2 mW/cm^2^ power density. The total energy dose after three hours of exposure is 2.16 J/cm^2^. The total energy received by cells per well in the 12-well plate is 7.56 J. The temperature is maintained at around 37 °C.

### 2.3. Western Blotting

Proteins were extracted from cells using LSB buffer containing a mixture of protease inhibitors (excluding EDTA) and phosphatase inhibitors. Perform BCA (ZJ102, Omni, Shanghai, China) quantification in accordance with the instructions of the kit. Equal amounts of proteins were separated by 10–12% SDS-PAGE gel electrophoresis and transferred onto PVDF membranes (Millipore, Bedford, MA, USA). The membrane was immersed in the blocking solution prepared with 5% milk for 2 h, washed with Tris Buffered Saline with Tween (TBST) three times for 5 min each time, and then incubated overnight at 4 °C. Primary antibodies: Opn3 (1:1000), p62 (1:2000), LC3B (1:3000), Pparα (1:1000), β-actin (1:5000), H1N1(1:2000). Subsequently, the membrane and the secondary antibody were incubated at room temperature for 1 h. Then wash three times with TBST, each time for 5 min. All bands were imaged using the ECL chemiluminescence system. Western blot original images can be found in Supplementary Materials.

### 2.4. Oil Red O Staining

After treating the cells with OA and light exposure, wash them twice with PBS, then fix them with 4% paraformaldehyde for 20 min, and wash them twice again with PBS to remove the excess paraformaldehyde. Subsequently, each well was treated with 60% isopropyl alcohol for 5 min and then discarded. Add the filtered oil red O working solution to the cells and stain them in the dark for 30 min, then wash them twice with distilled water. Finally, images were captured using an inverted microscope.

### 2.5. Real-Time PCR

Total RNA was extracted from cells using VeZol Reagent (R441-02-AA, Vazyme). The purity and concentration of RNA were measured using a Nanodrop-2000. Subsequently, the RNA was reverse-transcribed into an equal amount of cDNA using the SweScript All-in-One RT SuperMix for qPCR (G3337, Servicebio). Quantitative real-time PCR (RT-qPCR) was performed on a StepOne Real-Time PCR System using the 2× Universal Blue SYBR Green qPCR Master Mix (G3328-15, Servicebio). All procedures were conducted according to the manufacturer’s instructions. The primers were synthesized by Sangon Biotech Co., Ltd. (Shanghai, China). Gapdh was utilized as a normalization control. The primer sequences for the qPCR are provided in Supplementary Table S1.

### 2.6. Transfection and Establishment of Stable Cell Lines

The construction of lentiviral CRISPR-Cas9 vectors targeting genes was performed according to a standard experimental protocol. The detailed steps are as follows: Guide RNA (gRNA) was designed and synthesized, annealed, and then ligated into the vector via the BsmBI restriction sites. The gRNA sequences targeting Opn3 and p62 genes were obtained using an online design tool (e.g., CRISPR Design-http://chopchop.cbu.uib.no/, accessed on 26 November 2025) and are provided in Supplementary Table S2. For transient transfection and initial viral collection, HEK293T cells were seeded in 6-well plates and transfected with the plasmids required for lentivirus packaging using Lipofectamine 3000 (L3000015, Invitrogen, Carlsbad, CA, USA) according to the manufacturer’s instructions. To establish stable cell lines, the medium was changed 6 h after transfection and 3 mL of medium was added for continued culture. After 24 h, 2 mL of medium was added and the cells were cultured for one day. Subsequently, the lentiviral supernatant collected from the transfected HEK293T cells was centrifuged. Target cells were then infected in the presence of 5 μg/mL polybrene (107689, Sigma) and centrifuged at 3000 rpm for 1.5 h. Forty-eight hours post-transduction, the lentivirus-transduced Target cells were cultured in the presence of 2 μg/mL puromycin (ant-pr, Invivogen, Toulouse, France) for 2 weeks. Puromycin-resistant colonies were subsequently collected and subjected to further validation by Western blotting.

The procedures for gene knockdown experiments are similar. Using online shRNA design tools (e.g., Merck-https://www.sigmaaldrich.cn/CN/zh/semi-configurators/shrna?activeLink=productSearch, accessed on 26 November 2025) to generate primer sequences (Supplementary Table S3), followed by annealing and ligation into shRNA-specific vectors, with subsequent steps as described above.

### 2.7. BODIPY 493/503 Staining

Discard the culture medium from the cells, wash once with PBS, then add diluted BODIPY solution (1 μg/mL) for 15 min incubation protected from light. After removing the staining solution, add fresh PBS and observe under a fluorescence microscope using a 20× objective lens.

### 2.8. Triglyceride Assay

The triglyceride levels were measured using a Tissue/Cell Triglyceride Assay Kit (E1013, Applygen, Beijing, China), and the experimental procedures were strictly performed according to the manufacturer’s instructions. Experimental cells were treated with a specific volume of lysis buffer, followed by centrifugation. An appropriate volume of supernatant was then mixed with the working solution in a 96-well plate. After incubation at 37 °C for 15 min, absorbance was measured at a wavelength of 550 nm. The results were quantified by using a standard curve and the protein BCA quantitative method for extrapolation.

### 2.9. Cholesterol Assay

Total cholesterol levels were determined using a Total Cholesterol Assay Kit (E1015, Applygen), and the experimental procedures were strictly performed according to the manufacturer’s instructions. Experimental cells were lysed with a defined volume of lysis buffer and centrifuged. A precise volume of supernatant was combined with the working solution in a 96-well plate. Following a 15 min reaction at 37 °C, absorbance was recorded at 550 nm. The results were quantified by using a standard curve and the protein BCA quantitative method for extrapolation.

### 2.10. Viral Infection Assay

Vesicular stomatitis virus labeled with enhanced green fluorescent protein (VSV-GFP), EMCV, human influenza virus A/Puerto Rico/8/34 (H1N1) (PR8) were stored from our laboratory. Cells were seeded in culture plates and allowed to reach 60–70% confluence prior to experimentation. Wild-type AML12 cells were infected with diluted viral suspensions at the following multiplicities of infection: GFP-VSV (1:500), EMCV (1:10,000), and H1N1 (1:10,000). Similarly, Opn3-knockout AML12 cells were infected with VSV (1:500), EMCV (1:10,000), and H1N1 (1:10,000). After 6 h of incubation in a cell culture incubator, the cells were exposed to 470–480 nm blue light for 3 h, followed by an additional 4 h of culture before subsequent analyses were performed.

### 2.11. PI Staining

After the designated treatments, cells were incubated with propidium iodide (PI) at a final concentration of 5 µg/mL in the appropriate binding buffer. After gentle mixing, the sample is incubated at room temperature for 30 min in the dark to prevent photobleaching, followed by image acquisition under a microscope.

### 2.12. Statistical Analyses

Data were analyzed using GraphPad Prism 9.0. All data were expressed as mean ± SD. Data were tested for a normal (Gaussian) distribution using Shapiro–Wilk normality test. For comparisons involving multiple groups, one-way ANOVA followed by Tukey’s or Dunnett’s multiple comparison test was used for multiple group comparisons. Comparisons between two groups were conducted using an unpaired *t*-test. A *p*-value of less than 0.05 was considered statistically significant.

## 3. Results

### 3.1. Degradation of Lipid Droplets in OA-Treated Cells Induced by Blue Light

**Figure 1:**
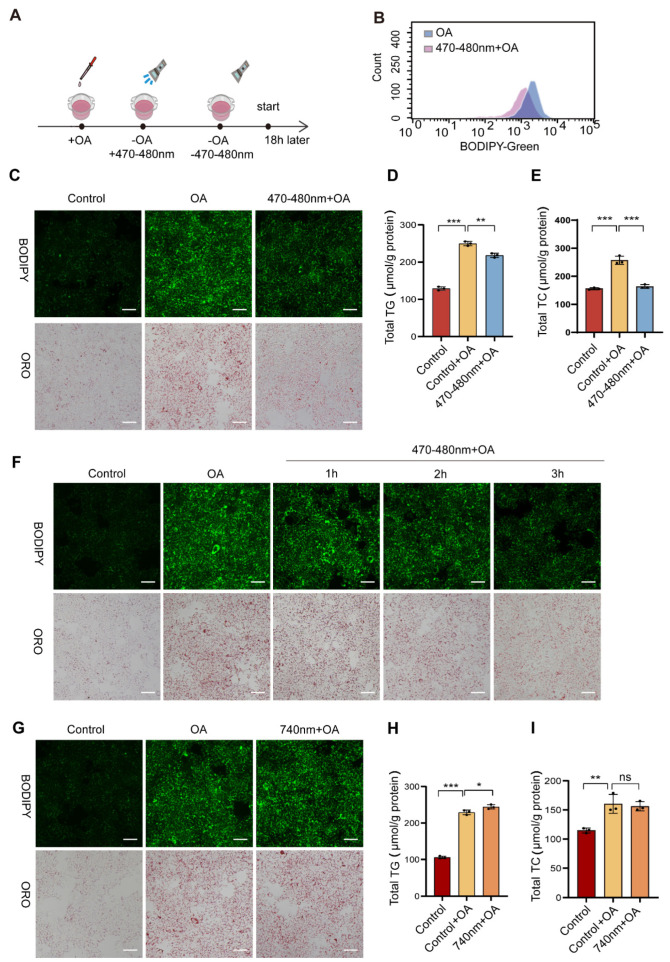
Degradation of lipid droplets in OA-treated cells induced by blue light. (**A**) Experimental procedure for exposing AML12 to 470–480 nm blue light irradiation. (**B**) Flow cytometric analysis of lipid droplet content in oleic acid (OA)-induced cells with or without 470–480 nm blue light treatment, *n* = 3. (**C**) Representative micrographs of BODIPY 493/503 and Oil Red O staining in cells treated with 470–480 nm blue light, Scale bar = 100 μm, *n* = 3. (**D**,**E**) Cellular triglyceride (TG) and total cholesterol (TC) levels in AML12 cells under 470–480 nm blue light and OA co-treatment, *n* = 3. (**F**) Microscopy images of BODIPY 493/503 and Oil Red O staining were used to analyze the effects of different 470–480 nm blue light exposure times on lipid droplets, Scale bar = 100 μm, *n* = 3. (**G**) Micrographs of BODIPY 493/503 and Oil Red O staining in cells treated with 740 nm red light, Scale bar = 100 μm, *n* = 3. (**H**,**I**) TG and TC content in AML12 cells following treatment with 740 nm red light and OA, *n* = 3. Data are shown as mean ± *SD*; ns: not significant, ^*^ *p* < 0.05, ^**^ *p* < 0.01, ^***^ *p* < 0.001. *n* = 3 biologically independent replicates per experiment, with three technical replicates per group.

In this study, the AML12 was used as the main model system. Lipid droplet accumulation was induced by oleic acid (OA) treatment. In the experimental design, cells were first treated with OA to promote lipid droplet accumulation. The medium was then replaced with fresh medium, and the cells were immediately exposed to blue light. After irradiation, the cells were cultured for another 18 h before analysis (Figure 1A). Flow cytometry analysis revealed that OA-treated cells exposed to blue light exhibited significantly lower fluorescence intensity compared to unexposed OA-treated controls, indicating reduced lipid droplet content (Figure 1B). To further evaluate lipid droplet degradation, BODIPY and Oil Red O (ORO) staining were also performed following blue light exposure (Figure 1C). OA-treated cells (Control + OA) showed intense green fluorescent staining and red staining, indicating substantial lipid droplet accumulation. In contrast, blue light exposure markedly reduced lipid droplets. Similarly, the changes in intracellular triglyceride and cholesterol levels were consistent with the observations described above (Figure 1D,E). To determine the optimal duration of light exposure, different treatment times were tested. The results showed that a three-hour light exposure period produced a significant reduction in lipid droplets (Figure 1F). To further investigate the specificity of blue light, experiments were conducted with 740 nm red light irradiation (Figure 1G–I). After three hours of red light exposure, OA-treated cells showed no reduction in lipid droplet staining with either BODIPY or Oil Red O. Additionally, TG and TC levels remained unchanged relative to the non-irradiated group. These findings indicate that 740 nm red light did not induce lipid droplet degradation.

### 3.2. Opn3 Mediates the Degradation of Lipid Droplets Induced by Blue Light

**Figure 2:**
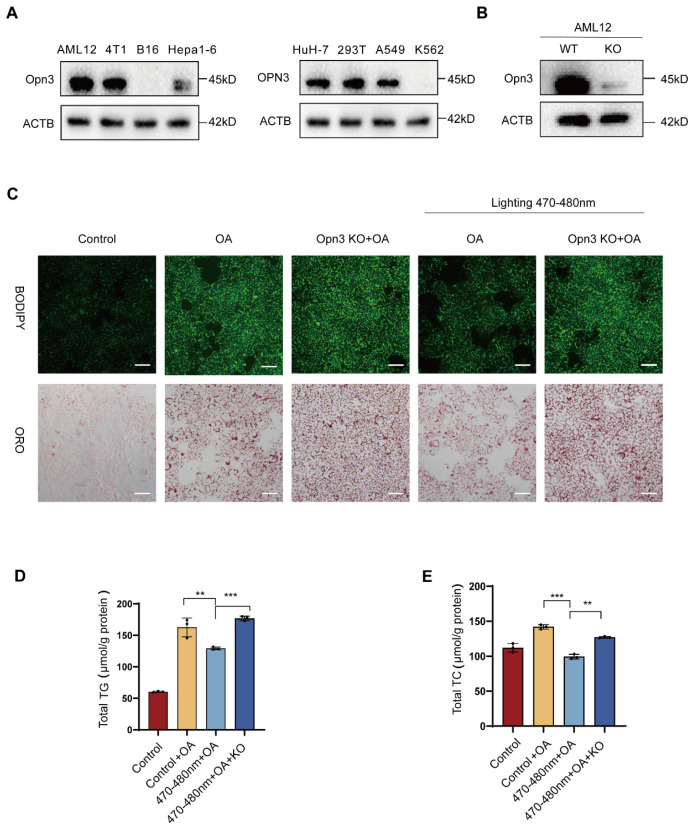
Opn3 mediates the degradation of lipid droplets induced by blue light. (**A**). WB is used to detect the expression levels of Opn3 protein in different cells, *n* = 3. (**B**). WB assay was used to detect the protein expression of Opn3 in the control group and the AML12 Opn3 KO group, *n* = 3. (**C**). Microscopic images of BODIPY 493/503 and oil red staining, scale bar = 100 μm, *n* = 3. (**D**,**E**). Content of total TG and TC in AML12 cells and AML12 Opn3 KO cells treated with 470–480 nm blue light and OA, *n* = 3. Data are shown as mean ± *SD*, ^**^ *p* < 0.01, ^***^ *p* < 0.001, *n* = 3 biologically independent replicates per experiment, with three technical replicates per group.

To investigate the role of Opn3 in blue light-induced lipid droplet degradation, we examined its expression levels in multiple murine and human cell lines, including AML12, 4T1, B16, Hepa1-6, Huh7, 293T, A549, and K562. Based on this screen, AML12 cells were selected for subsequent studies (Figure 2A). To test the necessity of Opn3 in blue light-induced lipid droplet degradation, this study established an Opn3-knockout (KO) AML12 cell line (Figure 2B). Subsequently, Cells were divided into four experimental groups to compare the response of wild-type and Opn3 KO cells to blue light. Blue light irradiation markedly reduced lipid droplet content in wild-type AML12 cells. In contrast, this effect was abolished in Opn3 KO cells, which resisted light-induced degradation (Figure 2C). To further quantify the degradation efficiency, we measured total TG and TC levels. Consistent with the results observed under the microscope, blue light irradiation significantly reduced TG and TC content in wild-type cells, but not in the Opn3 KO cells (Figure 2D,E). To further validate the findings, this study also conducted experiments using A549 cells and obtained consistent results (Figure S1A–D). In A549 cells, the lipid-reducing effect of blue light remained evident, but was abolished by OPN3 KO. These results indicate that Opn3 is essential for blue light-induced lipid droplet degradation.

### 3.3. Gene Changes in Blue Light and OA-Treated Cells After Opn3 Knockout

**Figure 3:**
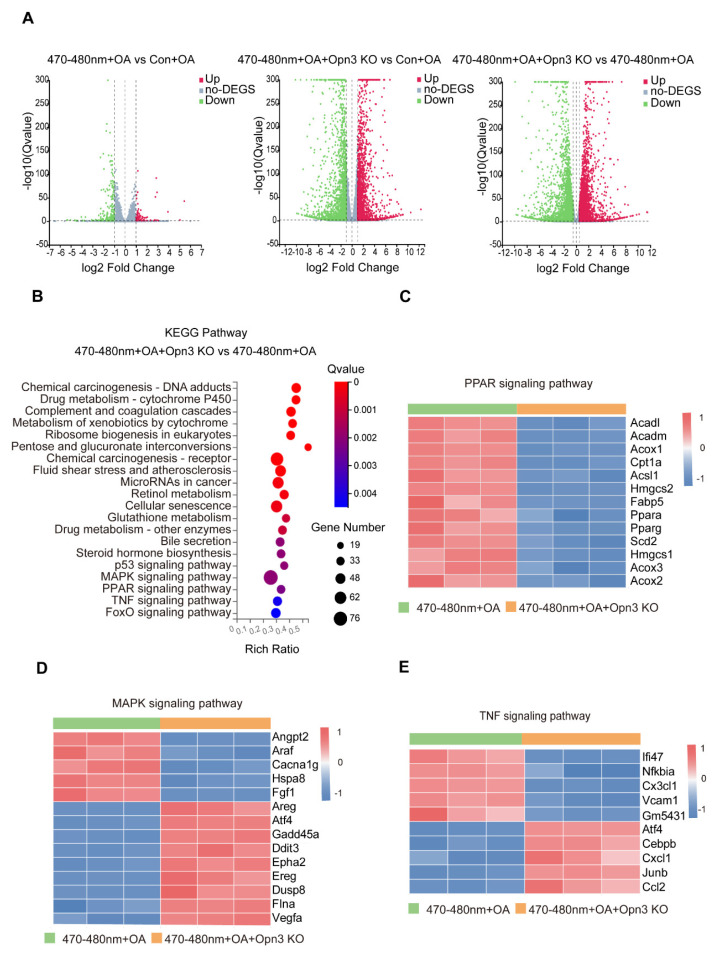
Gene changes in blue-light and OA-treated cells after Opn3 knockout. (**A**). Volcano plots of differentially expressed genes in the Con + OA group and the 470–480 nm + OA group (*n* = 3), the Con + OA group and the 470–480 nm + OA + Opn3 KO group, and the 470–480 nm + OA group (*n* = 3) and the 470–480 nm + OA + Opn3 KO group (*n* = 3). (**B**). KEGG pathway enrichment analysis of differentially expressed genes in AML12 Opn3 KO and AML12 treated with 470–480 nm blue light and OA (*n* = 3). (**C**). Heatmap of PPAR signaling pathway. (**D**). Heatmap of MAPK signaling pathway. (**E**). Heatmap of TNF signaling pathway. *n* = 3 biologically independent replicates per experiment, three technical replicates per group.

In order to understand the molecular mechanism of blue light-induced lipid droplet degradation, we performed transcriptome analysis of mouse hepatocytes. Compared to the Con + OA group, blue light irradiation (470–480 nm + OA) resulted in 742 downregulated and 521 upregulated genes. In the 470–480 nm + OA + Opn3 KO group and the Con + OA group, a total of 3367 genes were upregulated and 3530 genes were downregulated. And a direct comparison between the 470–480 nm + OA + Opn3 KO and 470–480 nm + OA groups identified 6269 differentially expressed genes, 3050 genes were upregulated and 3219 genes showed decreased expression (*p* value < 0.05 and log FC > 0.5) (Figure 3A). KEGG pathway analysis of the 470-480 nm + OA + Opn3 KO group versus the 470–480 nm + OA group revealed notable changes in the PPAR, MAPK, and TNF signaling pathways upon Opn3 knockout (Figure 3B). The PPAR signaling pathway, a key component in fatty acid metabolism, was significantly downregulated in Opn3 KO cells compared to the 470–480 nm + OA group. This was evidenced by reduced expression of approximately 13 pathway genes, including *Ppara*, *Pparg*, *Acox1*, *Cpt1a*, and *Fabp5* (Figure 3C). Similarly, heatmap analysis indicated that Opn3 deficiency also altered the MAPK and TNF signaling pathways. Within the MAPK pathway, which regulates cell proliferation and stress responses, the expression of key genes including *Araf*, *Fgf1*, *Atf4*, and *Vegfa* was significantly changed (Figure 3D). The TNF signaling pathway is involved in the processes of cellular inflammation and apoptosis. Among them, genes such as *Nfkbia*, *Ccl2*, and *Junb* also show differential expression (Figure 3E). These results suggest that Opn3 is probably involved in regulating lipid metabolism, modulates cellular stress and inflammatory responses.

### 3.4. Blue Light Induces Lipid Droplet Degradation Dependent on Pparα

**Figure 4:**
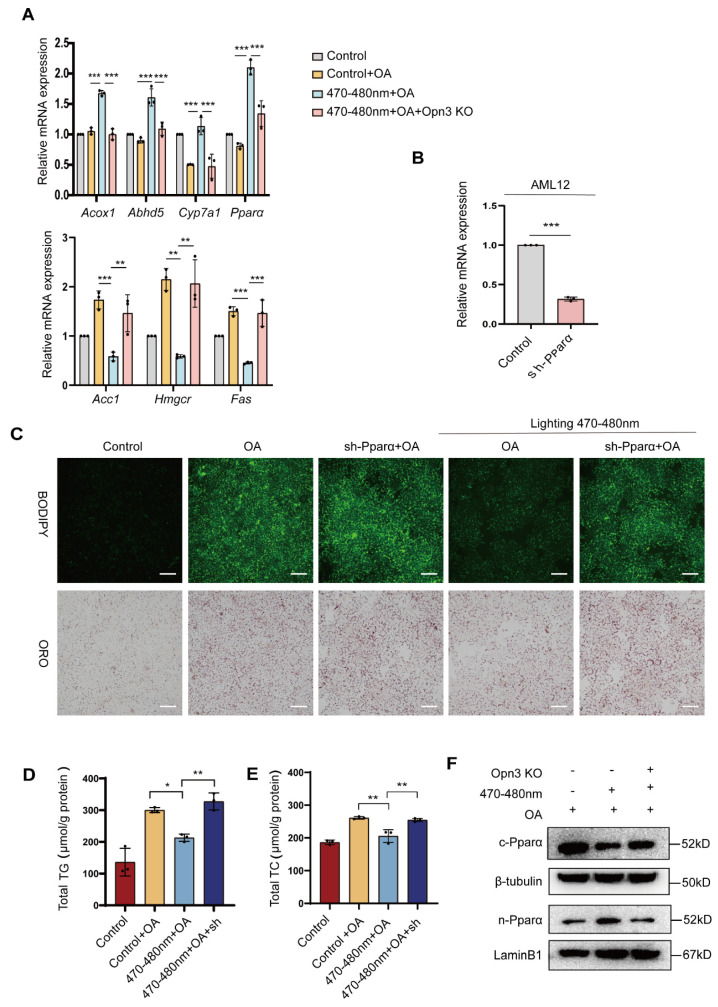
Blue light induces lipid droplet degradation dependent on Pparα. (**A**). RT-PCR analysis of key lipid metabolism enzymes, *n* = 3. (**B**). RT-PCR analysis of Pparα, *n* = 3. (**C**). Microscopy images of BODIPY 493/503 and oil red staining, scale bar = 100 μm, *n* = 3. (**D,E**). TG and TC contents of AML12 and AML12 sh-Pparα after 470–480 nm blue light and OA treatment, *n* = 3. (**F**). WB detection of Pparα expression in nuclear and cytoplasmic proteins of cells after exposure to 470–480 nm blue light and OA treatment, *n* = 3. All data are shown as the mean ± *SD*. ^*^ *p* < 0.05, ^**^ *p* < 0.01, ^***^ *p* < 0.001, *n* = 3 biologically independent replicates per experiment, with three technical replicates per group.

While Opn3 is essential for blue light-induced lipid droplet degradation, the downstream mechanisms remain unclear. Therefore, based on our transcriptomic data, we systematically compared the mRNA expression of key genes involved in both lipid degradation and synthesis pathways in blue light-irradiated AML12 cells. The experiment selected key genes in the lipid degradation pathway (such as *Acox1, Abhd5*, *Cyp7a1*, *Pparα*) and core regulatory factors in the lipid synthesis pathway (such as *Acc1*, *Hmgcr*, *Fasn*), and detected the changes in their transcriptional levels using RT-qPCR. The results showed that blue light exposure significantly upregulated the mRNA expression of lipid degradation-related genes. The most notable increase was observed in Pparα. In contrast, blue light failed to significantly alter the expression of these lipid degradation genes in Opn3 KO cells. At the same time, Expression of lipid synthesis pathway genes decreased after light exposure. However, Opn3 KO cells showed increased lipid synthesis (Figure 4A). Subsequent experiments were designed to determine whether 470–480 nm blue light-induced lipid droplet degradation depends on Pparα. We constructed cells with Pparα knocked down. RT-qPCR confirmed a significant reduction in Pparα mRNA levels (Figure 4B). BODIPY and Oil Red O staining revealed lipid droplet changes. In wild-type cells, OA treatment induced rapid lipid droplet accumulation, while blue light exposure significantly reduced staining intensity compared to the OA group. However, in Pparα KO cells, the ability of blue light to degrade lipid droplets was markedly inhibited (Figure 4C). Furthermore, TG and TC levels in Pparα-knockdown cells treated with both blue light and OA were significantly higher than the 470–480 nm + OA group (Figure 4D,E). Interestingly, study observed that blue light irradiation altered the nucleocytoplasmic distribution of Pparα in AML12 cells, increasing its nuclear localization and decreasing cytoplasmic levels. This effect was abolished upon Opn3 knockout (Figure 4F).

### 3.5. Blue Light Induces Lipid Droplet Degradation Through p62

**Figure 5:**
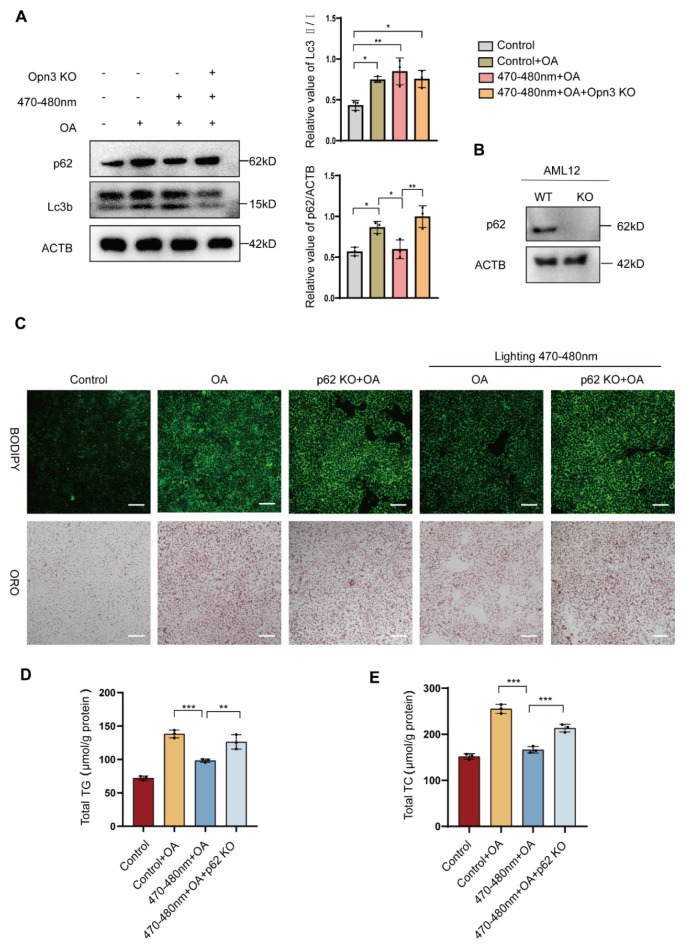
Blue light induces lipid droplet degradation through p62. (**A**): WB detects the protein expression of p62 and Lc3b in AML12 and Opn3 KO group after exposure to 470–480 nm blue light and OA treatment, *n* = 3. (**B**): WB assay was used to detect the protein expression of p62 in the AML12 group and the p62 KO group, *n* = 3. (**C**): Microscopic images of BODIPY 493/503 and oil red staining, scale bar = 100 μm, *n* = 3. (**D,E**): TG and TC contents of AML12 cells and p62 KO cells after OA treatment at 470–480 nm Blue light, *n* = 3. All data are shown as the mean ± *SD*. ^*^ *p* < 0.05, ^**^ *p* < 0.01, ^***^ *p* < 0.001, *n* = 3 biologically independent replicates per experiment, with three technical replicates per group.

Lipid droplets are key structures for storing neutral lipids in cells, and their dynamic balance is also regulated by autophagy. To investigate whether the mechanism of blue light-induced lipid droplet degradation involves autophagy, we performed Western blot analysis. In wild-type cells, blue light exposure alone significantly increased the LC3B-II/I ratio only in OA-treated cells, while p62 protein levels were elevated. Combined treatment with blue light and OA further enhanced the LC3B-II/I ratio and reduced p62 expression, indicating enhanced autophagic flux. However, in Opn3 KO cells, blue light exposure resulted in an increased LC3B-II/I ratio and a concurrent rise in p62 levels compared to the control group (Figure 5A), indicating that autophagy was inhibited. To evaluate autophagic flux, we used the autophagy inhibitor chloroquine (CQ). In cells treated with blue light and OA, CQ addition resulted in greater LC3B-II accumulation and elevated p62 levels, validating that blue light enhances autophagic flux (Figure S2A). At the same time, the study found that light exposure can increase ATP synthesis in AML12 cells, but this increase does not occur in Opn3 KO cells (Figure S2B). To validate the role of autophagy in blue light-induced lipid droplet degradation, a stable p62 KO cell line was generated in AML12 cells (Figure 5B). The experiment also used BODIPY and Oil Red O staining to visually quantify changes in lipid droplet number. The results showed that in wild-type cells, the combination of OA and blue light treatment reduced lipid droplet quantity. However, p62 KO cells contained more lipid droplets than the 470–480 nm + OA group, showing no significant difference compared to OA treatment alone (Figure 5C). To quantitatively evaluate lipid droplet degradation efficiency, TG and TC levels were further examined. Blue light irradiation significantly reduced TG and TC content in cells. However, p62 KO cells exhibited significantly higher TG and TC levels compared to the 470–480 nm + OA group (Figure 5D,E).

### 3.6. Opn3 Mediates the Inhibition of the Replication of VSV, EMCV and H1N1 by Blue Light

The vesicular stomatitis virus (VSV), the encephalomyocarditis virus (EMCV), and the influenza virus (H1N1) are three types of pathogens that are of great significance in virology research and the development of antiviral treatment strategies. They can be used as models for broad-spectrum antiviral research.

**Figure 6:**
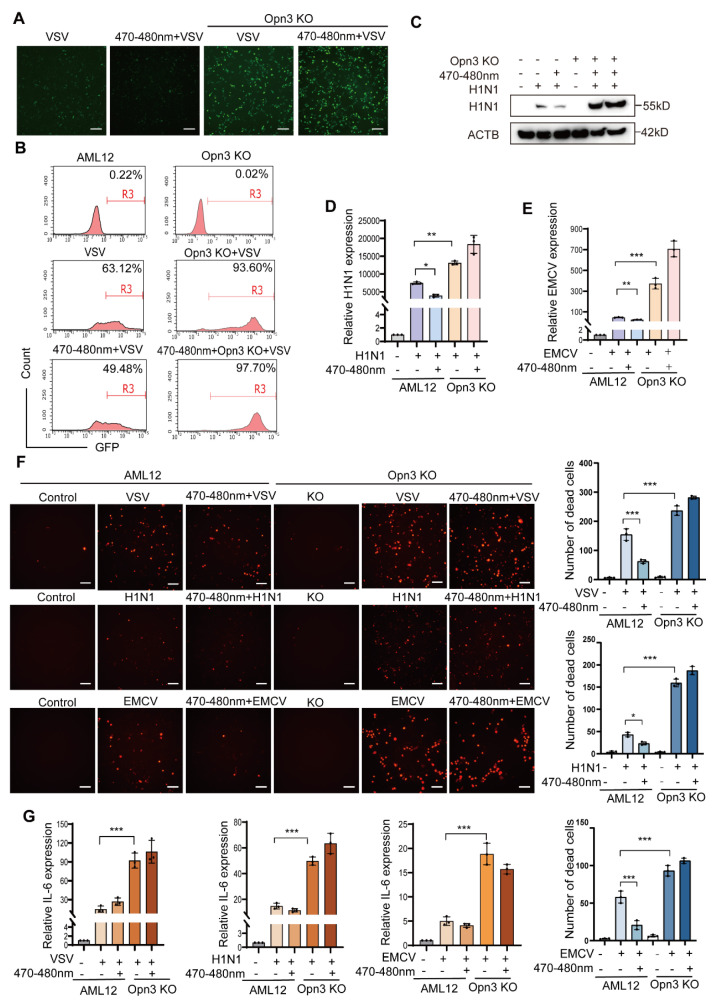
Opn3 mediates the inhibition of the replication of VSV, EMCV and H1N1 by blue light. (**A**). Fluorescence image of GFP-VSV infection in AML12 cells captured by a microscope, scale bar = 100 μm, *n* = 3. (**B**). Flow cytometry to detect the virus infection efficiency of GFP-VSV, *n* = 3. (**C**). WB to detect the expression of H1N1 protein in the AML12 and Opn3 KO cells after blue light and H1N1 virus treatment, *n* = 3. (**D**). RT-PCR analysis of H1N1, *n* = 3. (**E**). RT-PCR analysis of EMCV, *n* = 3. (**F**). PI staining of different viruses infecting AML12 and Opn3 KO cells captured by a microscope, scale bar = 100 μm, *n* = 3. (**G**). RT-PCR analysis of IL-6 in different virus-infected AML12 and Opn3 KO cells, *n* = 3. All data are shown as the mean ± *SD*. ^*^ *p* < 0.05, ^**^ *p* < 0.01, ^***^ *p* < 0.001, *n* = 3 biologically independent replicates per experiment, with three technical replicates per group.

During the research, we unexpectedly discovered that blue light irradiation inhibited the replication of VSV, EMCV, and H1N1 viruses. To quantify VSV infection, we used a GFP-tagged virus. Cells that have been successfully infected by the virus can emit green fluorescence. Decreased green fluorescence in AML12 cells confirmed that blue light significantly reduced infection. However, this protective effect was absent in Opn3 KO cells, which showed higher infection rates than wild-type AML12 cells (Figure 6A). Flow cytometry analysis supported these findings, after exposing AML12 cells to blue light, the fluorescence intensity of the cells infected by VSV showed a significant downward trend compared to the control group that was not exposed to light. In contrast, Opn3 KO cells showed no reduction upon irradiation and were more susceptible to infection than control group (Figure 6B). The VSV infection in A549 cells yielded consistent results (Figure S3A,B). We next examined EMCV infection. Western blot analysis showed that blue light exposure reduced viral infection rates in wild-type AML12 cells after EMCV infection. In contrast, Opn3 KO cells exhibited high infection rates (Figure 6C). Subsequently, viral mRNA expression of both H1N1 and EMCV was compared in Opn3 KO and wild-type cells under light and no light conditions. The results were consistent with the Western blot data (Figure 6D,E). Cell viability after infection with the three viruses was assessed by PI staining. Consistent with the infection data, Opn3 KO cells showed a sharp increase in PI-positive cells following viral challenge, which was not alleviated by blue light exposure. In contrast, wild-type cells exhibited moderate cell death that was effectively suppressed by light treatment (Figure 6F).

**Figure 7:**
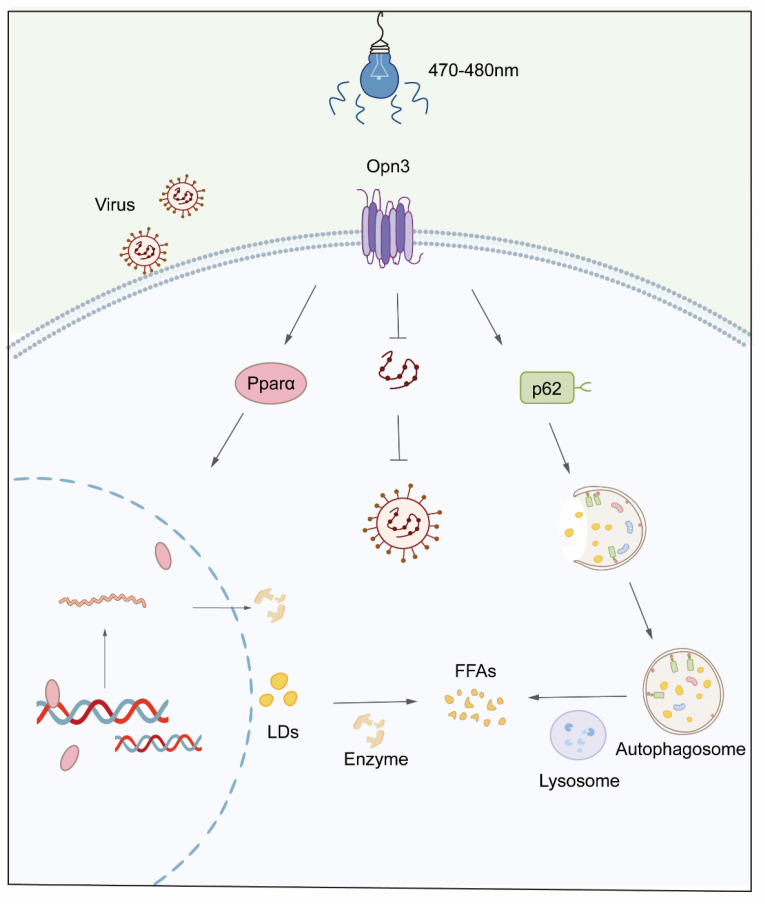
A model of blue-light-mediated Opn3 degradation of lipid droplets and the antiviral mechanism.

To assess the inflammatory response to infection, we measured the mRNA expression of the key pro-inflammatory cytokine *IL-6*. Compared with the AML12 cells, viral infection significantly induced the expression of *IL-6* in Opn3 KO cells. During the viral infection process, the amount of inflammatory factors expressed by the wild-type AML12 was significantly lower than that in the Opn3 KO cells, and this result was consistent in three different virus infection experiments. A similar trend was observed for *IL-6* production following VSV infection of A549 cells (Figure S3C). Based on the above results, the replication of VSV, EMCV and H1N1 is closely related to Opn3 and blue light. (Figure 7).

## 4. Discussion

This study combined light exposure with lipid droplet metabolism. The results show that 470–480 nm blue light effectively reduced OA-induced lipid droplets in cells. A comparison of different exposure times (1 h, 2 h, 3 h) revealed a time-dependent degradation rate, with a marked increase in degradation efficiency observed in the 3 h group. Notably, the 740 nm red light control caused no lipid droplet reduction, ruling out non-specific effects like heat or phototoxicity. Subsequent experiments using Opn3 KO cells showed strong resistance to the blue light effect, confirming that Opn3 is necessary for the light signal. To investigate the mechanism by which Opn3 mediates lipid droplet degradation, we analyzed the mRNA expression of key genes involved in lipid synthesis and breakdown pathways through transcriptomic analysis. Following blue light exposure (470–480 nm), the expression of lipid catabolism genes was significantly upregulated. Conversely, this effect was reversed in Opn3 KO groups under identical conditions. In contrast, the expression of lipid synthesis factors decreased after light exposure, but increased upon Opn3 KO. Among the changes, Pparα was particularly prominent. As a central transcription factor regulating lipid oxidative metabolism, it promotes fatty acid β-oxidation by activating the expression of key enzymes involved in fatty acid breakdown [23]. The study identified Pparα as a key downstream effector in blue light-induced lipid droplet degradation, whose absence blocked the photolytic effect on lipids. As evidenced by the results in Figure 2, Pparα knockdown produced a phenotype similar to Opn3 KO, effectively resisting the blue light effect. Specifically, Pparα knockdown prevented the degradation of lipid droplets mediated by blue light through Opn3. This indicates that both molecules operate within the same pathway, with Pparα functioning as a component in the downstream of Opn3. Beyond conventional lipolysis, lipid droplets can also be degraded via autophagy. We therefore investigated whether this pathway contributes to the light-induced effect. OA treatment alone increased the LC3B-II/I ratio and P62 levels, suggesting autophagic initiation with subsequent flux blockade. Blue light irradiation following OA treatment elevated the LC3B-II/I ratio and significantly reduced p62, indicating that blue light reduces lipid droplets through autophagy. In Opn3 KO cells, blue light irradiation increased the LC3B-II/I ratio and again elevated p62 levels. The downstream of autophagy was blocked, while lipid droplet autophagy was inhibited. The Western blot analysis was consistent with the other experimental results, and further demonstrated that blue light and Opn3 facilitate lipid degradation through the pathway of lipophagy.

VSV and EMCV, as well as the H1N1 virus, are well-known model viruses that have made significant contributions in academic research and the medical field. This study reveals a central role for Opn3 in light-induced antiviral defense. Blue light, acting through an Opn3-dependent mechanism, significantly suppressed the replication of diverse viruses including VSV, EMCV, and H1N1, while also reducing virus-induced cell death and inflammation. Conversely, Opn3 deficiency enhanced viral replication. This study found that blue light significantly inhibits the replication of multiple viruses through Opn3. It is noteworthy that a substantial body of research has demonstrated a correlation between lipids and viral replication, where the presence of lipids can accelerate infection by certain viruses and promote inflammatory responses. Lipid droplets can serve as platforms facilitating viral assembly and replication, including for viruses such as Zika virus [22], Junín virus [24], and Rabies virus [25]. Cholesterol has also been shown to promote the replication of Influenza A virus (IAV) and accelerate infection [26]. Therefore, we propose a possible mechanism for the observed blue light-mediated inhibition of viral replication via Opn3: blue light-driven lipid droplet breakdown and fatty acid oxidation may rapidly deplete the lipid resources essential for viral assembly, thereby restricting viral replication.

Pparα is a key transcriptional regulator of fatty acid metabolism. It can be activated not only by endogenous ligands, including fatty acids and eicosanoids, but also by synthetic compounds like fibrates and thiazolidinediones, as well as indirectly through energy stress [27]. We propose three potential mechanisms for Opn3-mediated Pparα regulation. Firstly, as a nuclear receptor, Pparα operates within the cell nucleus. It acts as a transcription factor to promote the expression of fatty acid enzymes. Therefore, Pparα requires the regulation of nuclear export proteins and import proteins to achieve stable expression in the nucleus. Li’s research team has found that hyodeoxycholic acid can block the interaction between RAN and the nuclear export receptor CRM1, leading to increased accumulation of Pparα in the nucleus [28]. Therefore, it is possible that Opn3 may promote Pparα activation—and thereby enhance lipid droplet breakdown under blue light—by directly or indirectly influencing CRM1 or other nuclear transport receptors. Secondly, Pparα must form a heterodimer with the retinoid X receptor (RXR) to regulate gene expression. It is possible that light activation of Opn3 could strengthen this dimerization process. Finally, transcriptome data from study shows significant differences in AMPK pathway activity between Opn3 KO cells and normal cells after blue light exposure. AMPK is a central regulator of cellular energy metabolism and lipid homeostasis, functioning by sensing changes in the AMP/ATP ratio. Therefore, another reasonable explanation is that Opn3 influences lipid metabolism through certain steps in the AMPK pathway. Supporting this view, Liu et al. have demonstrated that the AMPK/Pparα signaling pathway can regulate lipid metabolism in hepatocytes and ameliorate MAFLD [29]. Meanwhile, based on the re-analysis of transcriptomic data from the Control + OA and 470–480 nm + OA groups, we found that the expression of *Fads1* and *Fads2*, key genes catalyzing the biosynthesis of polyunsaturated fatty acids, was significantly upregulated upon blue light irradiation, suggesting that blue light may promote the synthesis of unsaturated fatty acids in cells. As polyunsaturated fatty acid metabolism is regulated by Pparα, this finding is highly consistent with the role of the “blue light-Opn3-Pparα” signaling axis identified in this study. At the same time, the expression of several key genes involved in saturated fatty acid synthesis, such as *Fas* and *Acc1*, was downregulated after blue light exposure, indicating inhibition of saturated fatty acid synthesis. This “up-down” pattern of gene expression collectively suggests that blue light may promote the formation of polyunsaturated fatty acids, thereby reducing oleic acid-induced lipid droplet accumulation. However, the specific pathways involved in this regulatory mechanism remain unclear. In the future, further research on the mechanism is needed to clarify the role of unsaturated fatty acids in lipid reduction under blue light.

As another major pathway for eliminating excess lipids, lipophagy also plays a vital role in the body [30,31]. One study found that blue light exposure increased the levels of LC3 and Beclin-1 in colon cancer cells. This light-triggered autophagy, which is mediated by the Opn3 photoreceptor pathway, resulted in the suppression of cancer cell growth [32]. Our study also indicates a correlation between Opn3-mediated lipid droplet breakdown and lipophagy under blue light. We observed an increase in LC3B protein levels and a reduction in p62degradation following light exposure. Since the absence of p62 impaired lipid droplet clearance after blue light, we propose that Opn3 may also reduce lipid accumulation through the lipophagy pathway.

## 5. Conclusions

In summary, this study collectively shows that 470–480 nm blue light promotes lipid droplet degradation via the Opn3-Pparα signaling axis and a p62-dependent autophagy pathway. Additionally, we found that blue light and Opn3 can influence viral replication and improve inflammatory responses. These findings thus suggest that Opn3 may offer a novel photobiological strategy to concurrently regulate lipid metabolism and enhance antiviral defense, providing potential avenues for developing non-invasive interventions against metabolic and viral diseases.

## Data Availability

The original contribution presented in this study are included in the article/Supplementary Materials. Further inquiries can be directed to the corresponding author.

## Supplementary Materials

The following supporting information can be downloaded at https://www.mdpi.com/article/10.3390/biom16010109/s1. Figure S1. OPN3 mediates the degradation of lipid droplets induced by blue light in A549. Figure S2. Blue light induces lipid droplet degradation through autophagy. Figure S3. OPN3 mediates the inhibition of the replication of VSV, EMCV and H1N1 by blue light in A549. Table S1. Primers of real-time qPCR used in this study. Table S2. Primers of KO used in this study. Table S3. Primers of shRNA used in this study. File S1. original-images.
