# Supplementary material for: Opn3 Drives Blue-Light-Induced Reduction in Lipid Droplets and Antiviral Defense
Source: Biomolecules. 2026 Jan 8;16(1):109. doi: 10.3390/biom16010109 (PMC12838999; doi:10.3390/biom16010109)

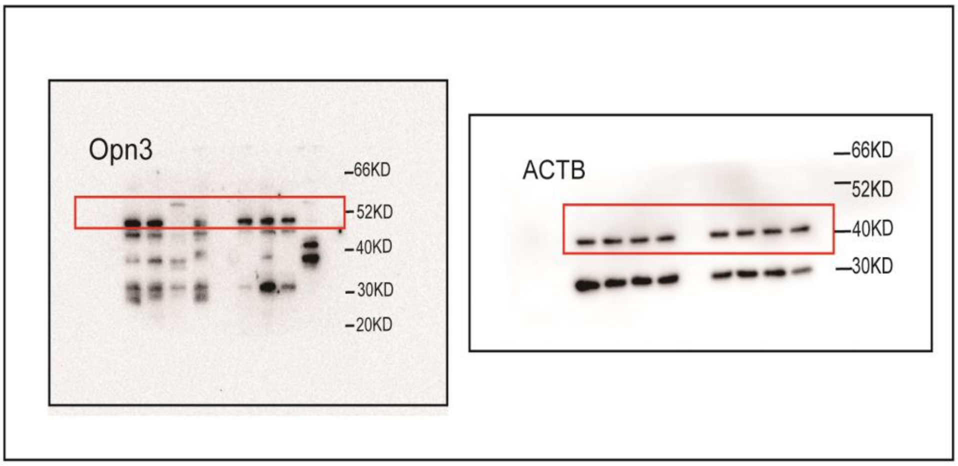


Figure2 A

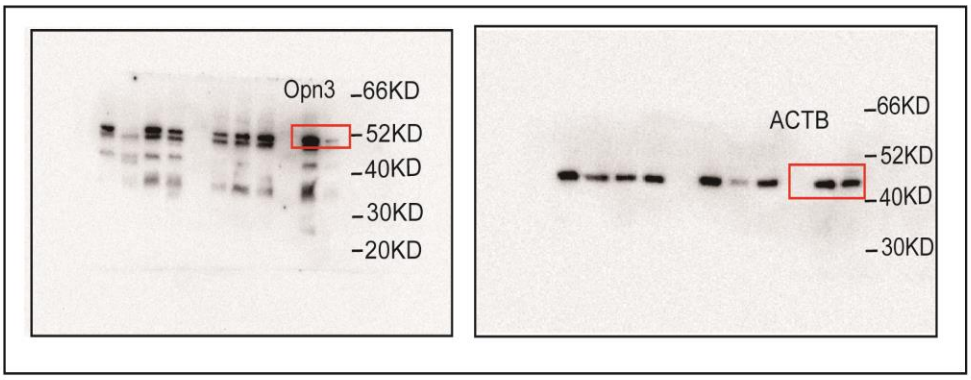


Figure2 B

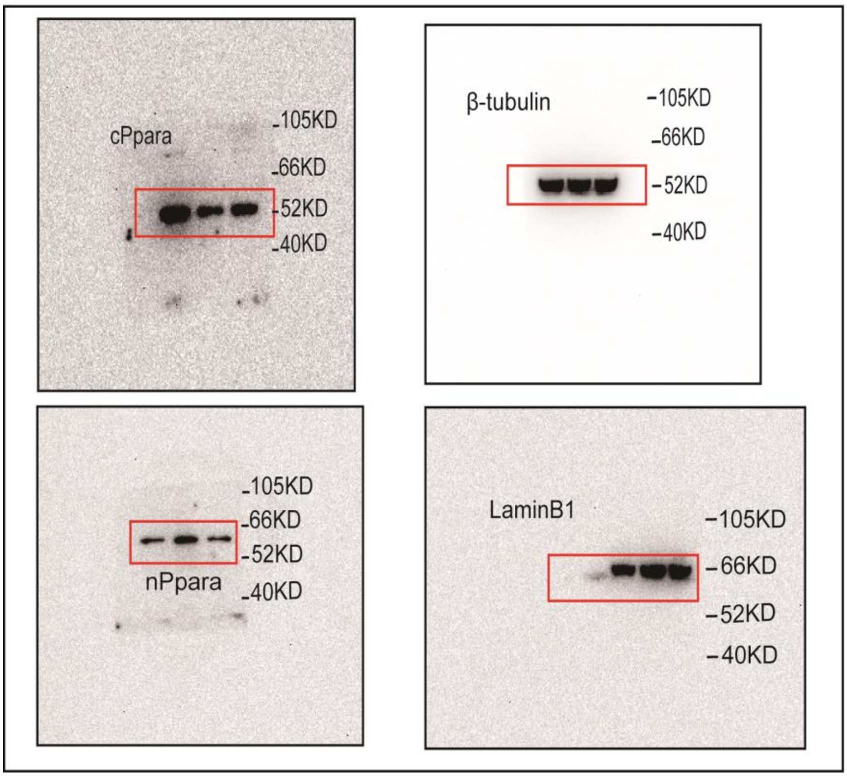


Figure4 F

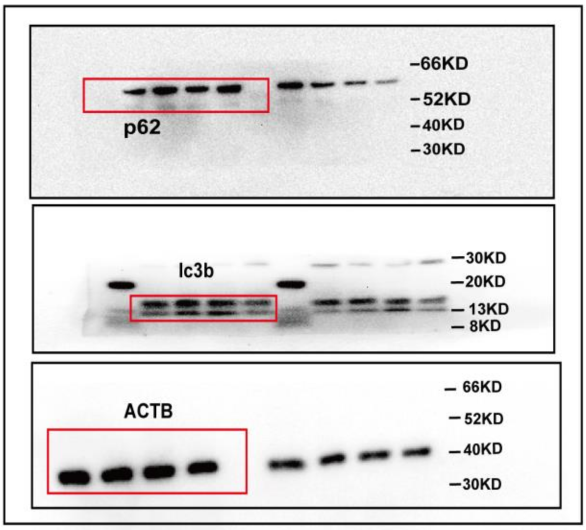


Figure5 A

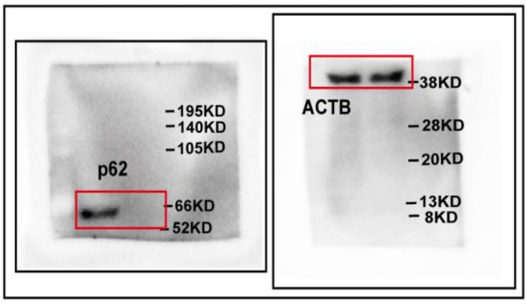


Figure5 B

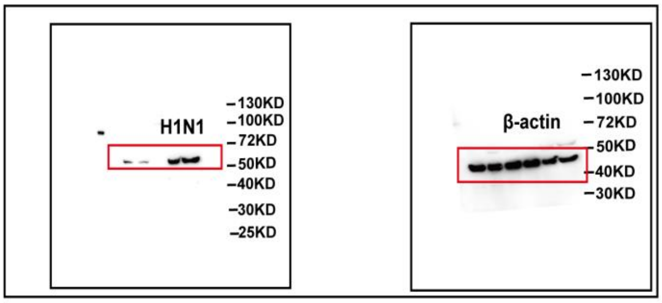


Figure6 C

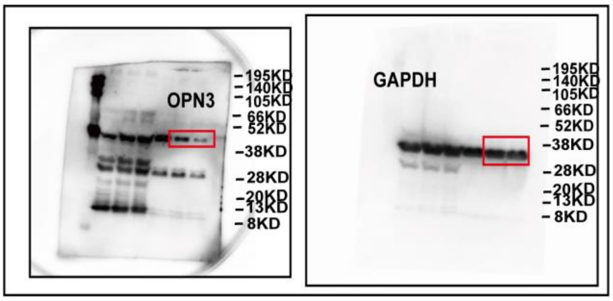


Supplementary Figure S1A

Supplementary Figure S2A

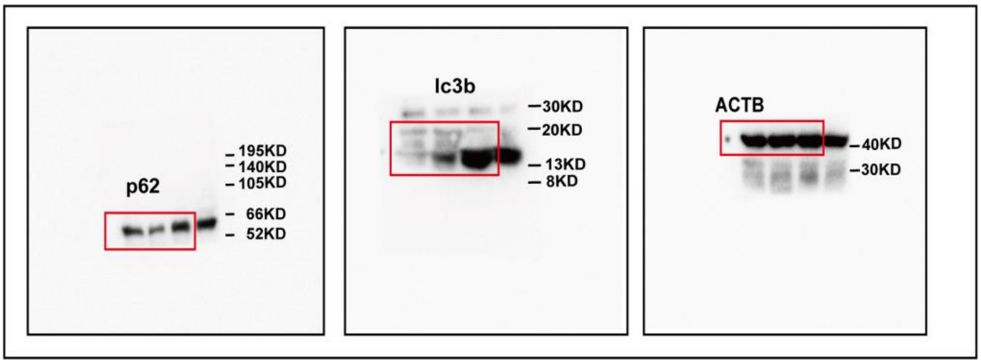

Supplement: Supplementary file 1 [file biomolecules-16-00109-s001.zip › File S1. original-images.pdf]
