# Supplementary material for: Opn3 Drives Blue-Light-Induced Reduction in Lipid Droplets and Antiviral Defense
Source: Biomolecules. 2026 Jan 8;16(1):109. doi: 10.3390/biom16010109 (PMC12838999; doi:10.3390/biom16010109)
Supplement: Supplementary file 1 [file biomolecules-16-00109-s001.zip › Revised Manuscript without Track Changes-Supplementary.pdf]

Supplementary Figure S1

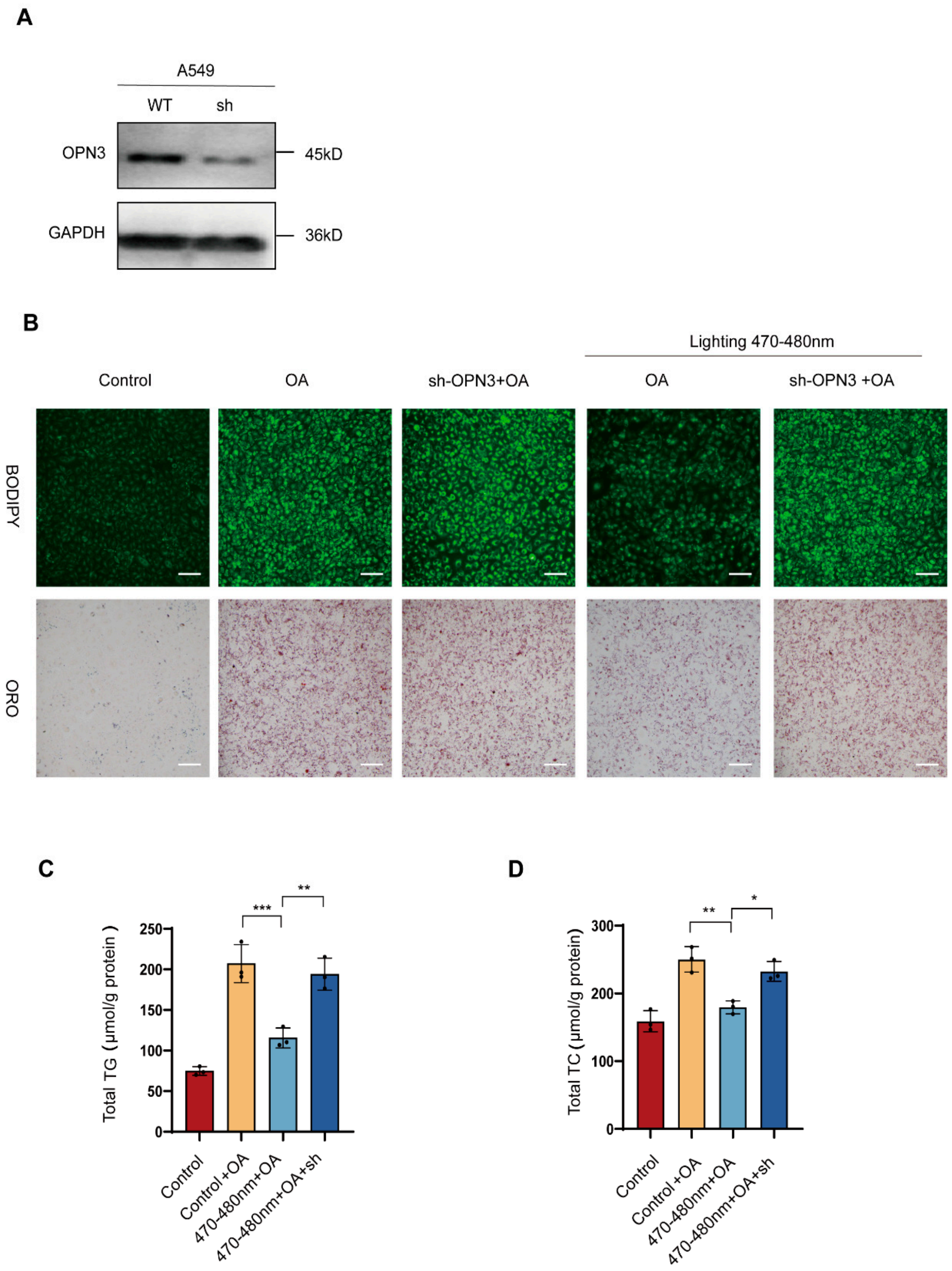

Supplementary Figure S1. OPN3 mediates the degradation of lipid droplets induced by blue light in A549

A. WB assay was used to detect the protein expression of OPN3 in the control group and the sh-OPN3 group, *n*=3.

B. Microscopic images of BODIPY 493/503 and oil red staining, scale bar =100 μm, *n*=3.

C-D. Content of total TG and TC in A549 cells and A549 sh-OPN3 cells treated with 470-480nm blue light and OA, *n*=3.

Data are shown as mean ± *SD*, $^{*}P < 0.05$, $^{**}P < 0.01$, $^{***}P < 0.001$, $n = 3$ biologically independent replicates per experiment, three technical replicates per group.

Supplementary Figure S2

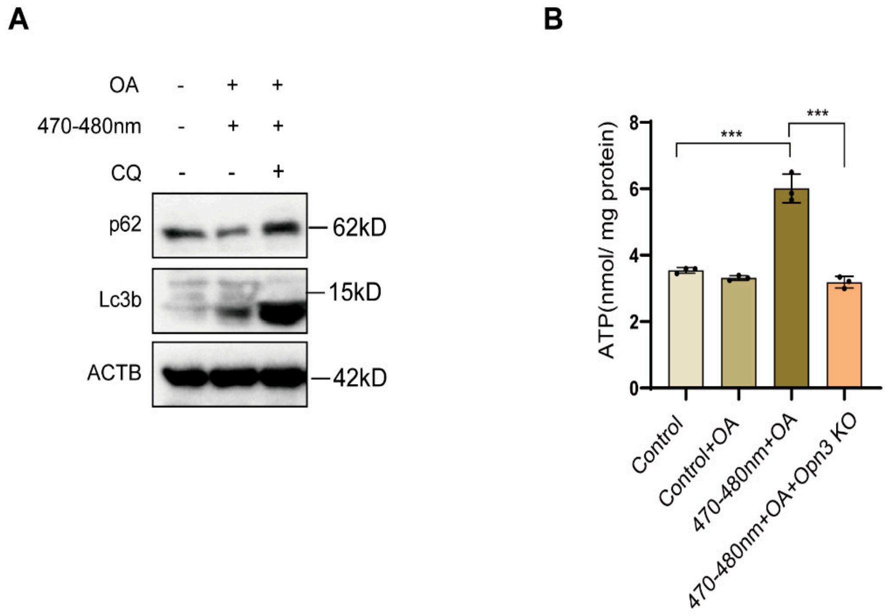

Supplementary Figure S2. Blue light induces lipid droplet degradation through autophagy

A. Change in ATP level, *n*=3.

B. WB detects the protein expression of p62 and Lc3b in AML12 after exposure to 470-480nm blue light, CQ and OA treatment, *n*=3.

Data are shown as mean ± *SD*, $^{***}P < 0.001$, $n = 3$ biologically independent replicates per experiment, three technical replicates per group.

Supplementary Figure S3

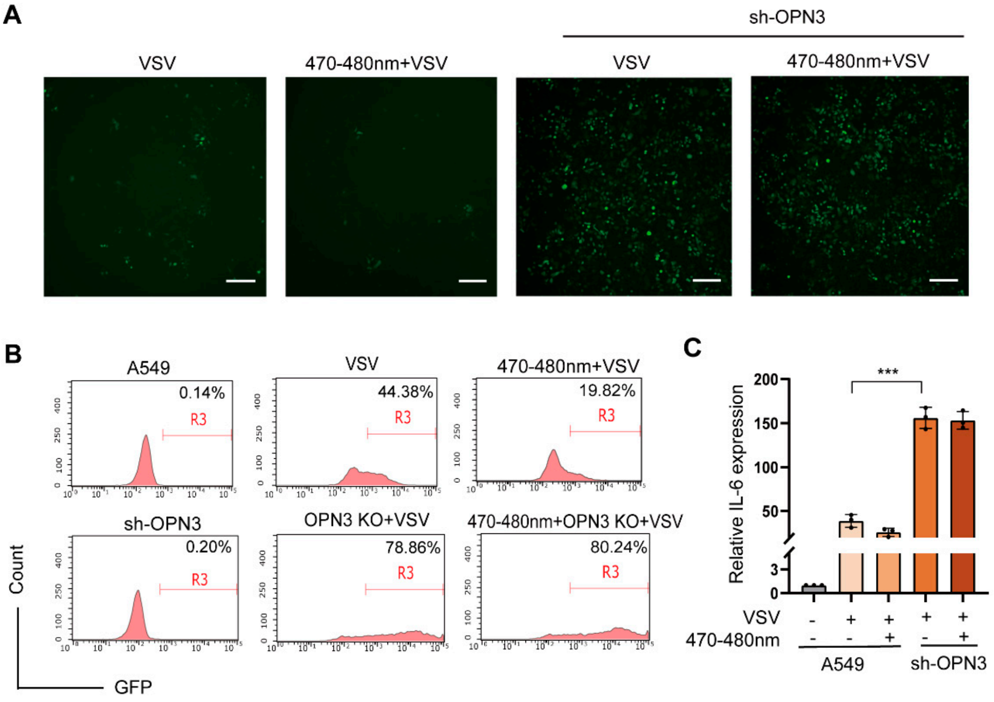

Supplementary Figure S3. OPN3 mediates the inhibition of the replication of VSV, EMCV and H1N1 by blue light in A549

A. Fluorescence image of GFP-VSV virus infection in A549 cells captured by a microscope, scale bar = 100 µm, *n*=3.

B. Flow cytometry to detect the virus infection efficiency of GFP-VSV virus, *n*=3.

C. RT-PCR analysis of *IL-6* in A549 and sh-OPN3 cells, *n*=3.

All data are shown as the mean ± *SD*. $^{***}P < 0.001$, $n = 3$ biologically independent replicates per experiment, three technical replicates per group.

.

Supplementary Table S1

Primers of real-time qPCR used in this study

Primers		Sequence
Acox1	F	AACATCATCACAGGGGCTCA
	R	TCAAAGGCATCCACCAAAGC
Cyp7a1	F	GGCATTTGGACACAGAAGCA
	R	TGGAGGTTTTGCATCATGGC
Pparα	F	AACATCGAGTGTCGAATATGTGG
	R	CCGAATAGTTCGCCGAAAGAA
Abhd5	F	AAGTCTAGTGCAGCGTTTGAG
	R	GGGCTCCAAAGATCACTGAAA
Acc1	F	CTCCCGATTCATAATTGGGTCTG
	R	TCGACCTTGTTTTACTAGGTGC
Hmgcr	F	AGAGCGAGTGCATTAGCAAAG
	R	GATTGCCATTCCACGAGCTAT
Fasn	F	GGCTCTATGGATTACCCAAGC
	R	CCAGTGTTCGTTCCTCGGA
Il-6	F	TAGTCCTTCCTACCCCAATTTCC
	R	TTGGTCCTTAGCCACTCCTTC
IL-6	F	TAGTCCTTCCTACCCCAATTTCC
	R	TTGGTCCTTAGCCACTCCTTC

Supplementary Table S2

Primers of KO used in this study

Primers		Sequence
Opn3	F	CACCGATGTACTCGGGGAACCGTAG
	R	AAACCTACGGTTCCCCGAGTACATC
p62	F	CACCGATGGTGGGCGATGTTCCCGC
	R	AAACGCGGGAACATCGCCCACCATC

Supplementary Table S3

Primers of shRNA used in this study

Primers		Sequence
Pparα	F	GATCCGCCCTTATCTGAAGAATTCTTCAA GAGAGAATTCTTCAGATAAGGGTTTTTTG
	R	AATTCAAAAAACCCTTATCTGAAGAATTC TCTCTTGAAGAATTCTTCAGATAAGGGCG
OPN3	F	GATCCGTCCATGCCAGAGTGATCATTCAA GAGATGATCACTCTGGCATGGATTTTTTG
	R	AATTCAAAAAATCCATGCCAGAGTGATCA TCTCTTGAATGATCACTCTGGCATGGACG
